# Spatial traces of trauma: changes in home perception in young individuals after the twin earthquakes

**DOI:** 10.3389/fpsyg.2026.1785005

**Published:** 2026-05-22

**Authors:** Mert Şen, Şevval Nur Şen

**Affiliations:** 1 Department of Classroom Education, Faculty of Education, İnönü University, Malatya, Türkiye; 2 Department of Fine Arts Education, Faculty of Education, İnönü University, Malatya, Türkiye

**Keywords:** disaster, home perception, place attachment, trauma and anxiety, twin earthquakes

## Abstract

This study examines how the meaning of “home” changed for young individuals residing in Malatya after the twin earthquakes centered on Kahramanmaraş on February 6, 2023. Designed as a phenomenological study, the research explores how perceptions of home shifted across spatial, emotional, and social dimensions before and after the disaster using a thematic analysis. The findings show that, before the earthquake, home was associated with comfort, trust, belonging, and sharing. After the earthquake, these meanings were replaced by anxiety, fear, uncertainty, loneliness, and loss. Home was no longer experienced as a secure and taken-for-granted space, but as a trauma-marked environment shaped by disrupted routines, broken trust, and memory-laden absence. The findings further suggest that the earthquake did not simply weaken attachment to place. It transformed the lived meaning of home and reconfigured place attachment as a fragile, trauma-inflected, and ambivalent experience of belonging. A clear rupture was particularly visible in the emotional process dimension of the tripartite place attachment model. Overall, the study highlights the need for recovery efforts that go beyond housing repair and include community-based social and psychological support aimed at restoring collective memory, everyday continuity, and a renewed sense of belonging.

## 1 Introduction

Today, increasing environmental disasters around the world have made it inevitable to understand how individuals cope with such crises. The rise of ecological concerns and the increasing number of individuals affected by disasters highlight the need to better understand how people perceive disaster risk and how they cope with it. In this context, it is important to understand the effects of disasters, which deeply affect young individuals’ spatial relationships, on individuals’ perceptions of home (Peng et al., 2020). The concept of home is not just a physical shelter for the individual, but carries multi-layered meanings in psychological, social and cultural terms. The concept of “housing,” which refers to the physical dimension of the structure, turns into “house” or “nest” as the individual makes sense of that space and attributes personal meanings to it (Babacan, 2021). In the literature, the social meanings of the home are generally discussed around themes such as safe haven, private space and spatial extension of the individual’s identity (Malett, 2004). Home is a concept that symbolizes the experiences, family and the deep structure of social relations (Babacan, 2021). Therefore, home is shaped as a psychological space through which individuals construct meaning by being equipped with values such as memories, relationships, daily routines and privacy beyond its physical boundaries. In other words, the concept of home turns into a psychological shelter as a result of the accumulation of the individual’s life experiences, feelings of security and belonging (Proshansky, 1978). In this context, individuals who instinctively evaluate space as a safe a place of safety reflect their identities on the place they live in and attach special meanings to it by developing emotional ties.

Existing literature draws attention to the emotional and social importance of the distinction between the concepts of dwelling and home, emphasizing the role of individuals’ sense of attachment to a place on positive outcomes such as happiness and psychological well-being (Mutlu and Tanrıverdi Kaya, 2020). However, the effects of large-scale disasters such as earthquakes on young individuals’ attachment to place and their perception of home remain a current area that needs to be investigated. In this context, examining the changes in individuals’ perceptions of the concept of home caused by the twin earthquakes centered in Kahramanmaraş on February 6, 2023, which researchers also directly experienced, has become an important research problem. Such a study has both academic and applied value in terms of understanding the spatial and psychosocial transformations of individuals after the disaster.

## 2 Theoretical frame: place attachment

Place attachment is defined as the emotional bond and psychological closeness that individuals establish with a particular place (Babacan, 2021). This bond is shaped not only by physical presence, but also by the quality of time spent in the place, the richness of accumulated memories, and the depth of established social relationships. In the literature, concepts such as place attachment, place identity, and place dependence are used to define the relationships that individuals establish with places (Jansen, 2020; Kutay Karaçor and Akçam, 2016). Each of these concepts expresses the different dimensions of meaning that individuals attribute to places and reveals different components of the relationship established with places.

Place attachment theory provides a comprehensive framework for describing the emotional, cognitive, and behavioral relationships an individual develops with a place. In this context, place attachment encompasses the strong emotional closeness and psychological feelings of belonging that an individual develops toward a particular place (Lee and Lin, 2022). On the other hand, place dependence is related to the importance that individuals attribute to the functional features of a particular space; the more adequate a space is in terms of meeting a person’s needs and goals, the more dependent the person becomes on that space (Anton and Lawrence, 2016). Place identity, on the other hand, emphasizes the symbolic meaning of place; this concept refers to the memories, emotions and ideas that a person shares in relation to a place becoming a part of the individual’s self-identity (Anton and Lawrence, 2016). Scannell and Gifford (2010) have presented a comprehensive framework that addresses place attachment in terms of three basic dimensions: person (who is attached?), process (how does attachment occur in terms of emotion, cognition, and behavior?), and place (what is the object of attachment?). This tripartite “person-process-place” framework provides a strong foundation for explaining the dynamic and multidimensional nature of place attachment.

The most important element of place attachment is the place itself with which the individual establishes a bond (Scannell and Gifford, 2010). The concept of home is one of the places where individuals experience place attachment most strongly. Because home is not only the physical structure in which the individual lives; it is also a psychological and emotional space where memories accumulate, a sense of trust is built, and identity and self-perception are shaped (Anton and Lawrence, 2014). In other words, the emotional meaning that the house carries beyond its physical features becomes the spatial counterpart of the individual’s self-identity and existential integrity (Proshansky et al., 1983). Therefore, the house is a psychologically and socially important place that carries the individual’s existential meanings and sense of identity, rather than a spatial phenomenon. In this context, the relationship between home and identity and home and existence is of critical importance in understanding the nature of the bond that individuals establish with space.

### 2.1 The relationship between home, memory and identity

The connection between home, memory and identity also plays an important role in the individual’s self-perception. Proshansky (1978) stated that the place where an individual lives is not only a coordinate, but also a space where they recognizes, expresses and remembers themselves. Memories, history and social experiences accumulated throughout life accumulate in physical spaces and form the identity and personal meaning of that place (Mutlu and Tanrıverdi Kaya, 2020). Through this process, individuals construct their sense of self and identity. In addition, the home is interwoven with the individual’s “self-universe” (Peng et al., 2020). This statement reveals that this palce is not just a shelter, but a component that plays an active role in the individual’s identity development. Many studies emphasize that a safe home environment is the foundation of the self, personal autonomy, stability and belonging (Twigger-Ross and Uzzell, 1996). This function of the home becomes even more apparent, especially during periods when stability is shaken. Stability, or in other words, the routine within the home, being shaken for various reasons also affects the individual’s relationship with the home.

### 2.2 The relationship of place attachment and disaster

Place attachment and the effects of disasters on places are one of the main research areas of environmental psychology (Smith and Cartlidge, 2011). Before disasters, place attachment can serve as an important protective factor in reducing disaster risk and increasing the resilience of communities. However, during disasters, the bonds between people and places can be disrupted or become dysfunctional. Reestablishing this bond requires a long-term process after the disaster (Ruiz and Hernández, 2014). With the occurrence of traumatic events such as natural disasters, the functions of the home for the individual, such as psychological security, belonging and identity, can be seriously weakened. In such cases, individuals’ spatial belongings are damaged; the emotional and psychological relationships they establish with their home are distrupded, and therefore, individuals’ self-identities can be shaken. For example, an individual who experiences an earthquake may experience a breakdown in their sense of identity, as well as their sense of security and belonging (Salcioglu et al., 2007). This is because when the spaces that an individual sees as “home” are damaged, their self-perception may also be negatively affected. Studies on the psychosocial effects of disasters reveal that the sense of security and control of individuals who experience trauma are severely shaken, and that those who experience a disaster are unable to perceive their homes in the same way as before (Manzo and Devine-Wright, 2020; Asadi et al., 2021; Scannell and Gifford, 2010; Twigger-Ross and Uzzell, 1996).

The disruption in place attachment experienced in the post-disaster process is closely related to the change in individuals’ environmental perception. Immediately after a disaster, individuals tend to re-evaluate spatial features that they previously considered ordinary or commonplace, and attribute higher importance to features such as the physical durability and structural safety of the home (Reza et al., 2024). In societies experiencing a large-scale earthquake, every spatial dimension in the home and its immediate surroundings is re-interpreted. As revealed in the study conducted by Asadi et al. (2021) on post-earthquake shelter environments, the language used by participants to describe the space differed in parallel with the emotional change they experienced after the disaster (Asadi et al., 2021). These findings indicate that large-scale traumatic events such as earthquakes restructure individuals’ environmental perceptions.

A significant portion of the existing literature focuses on place attachment and perceived disaster risk in post-disaster regions (Bonaiuto et al., 2016; Swapan and Sadeque, 2021; Shobkolaie et al., 2024; Jansen, 2020; Reza et al., 2024). According to the literature, place attachment of individuals is damaged after large-scale disasters; and the recovery and reconstruction processes of individuals take a long time. However, how the “perception of home”, which is closely related to place attachment, is transformed after large-scale disasters (especially twin earthquakes) is an important research question that has not yet been sufficiently answered in the literature.

While foundational models of place attachment, including Scannell and Gifford (2010) person–process–place framework, provide an important conceptual basis for understanding how bonds with place are organized and expressed, our focus on post-earthquake home perception suggests the need for further theoretical elaboration in disaster contexts. Existing scholarship has been highly effective in showing that place attachment is multidimensional and involves affective, cognitive, and behavioral processes shaped by both individual and collective meanings (Scannell and Gifford, 2010). At the same time, phenomenological approaches remind us that attachment is embedded in the broader lived experience of place, including routine, embodied inhabitation, and the taken-for-granted structure of everyday life (Seamon, 2014). In the aftermath of a catastrophic earthquake, this lived structure is not simply weakened. It is disrupted and reconstituted through fear, loss, memory, and existential insecurity. In this sense, the present study does not reject established place attachment theory. Instead, it extends it by showing that, in disaster contexts, home may no longer function as a stable site of security and belonging, but can become an ambivalent space marked by rupture, vulnerability, and traumatic meaning. In this context, the current study aims to address this theoretical need by examining how young people who experienced the twin earthquakes reinterpret the meaning of home.

### 2.3 February 6 Kahramanmaraş twin earthquakes and current study

It is reported that approximately 20% of earthquakes with a magnitude greater than 7.5 worldwide appeared in the literature as twin/doublet earthquakes. In the discipline of seismology, doublet earthquakes are defined as two consecutive main shocks that occur close to each other in time and space, exhibit similar seismic wave characteristics, and are close to in magnitude. The two consecutive major earthquakes centered in Kahramanmaraş on February 6, 2023 are also considered doublet earthquakes. The first earthquake occurred in the Pazarcık district of Kahramanmaraş with a magnitude 7.7 Mw; the second earthquake centered in Elbistan was recorded approximately 9 h later with a magnitude of 7.6 Mw (Sever, 2024). These earthquakes caused heavy damage and many casualties in 11 different provinces, primarily in Malatya, as well as Adana, Adıyaman, Diyarbakır, Elazığ, Gaziantep, Hatay, Kilis, Osmaniye and Şanlıurfa (Arslankoç et al., 2024). According to official data, 53,537 people lost their lives, 107,213 people were injured, approximately 14 million people were directly affected and approximately 1.5 million people were left homeless due to these earthquakes throughout Turkey. In addition, it was determined that 35,355 buildings in the region were completely destroyed, 17,491 buildings required urgent demolition, 179,786 buildings were severely damaged, 40,228 buildings had moderate damage and 431,421 buildings had minor damage (TMMOB, 2023).

After the earthquake, not only the public but also many individuals who directly experienced the disaster, including researchers, had to leave their homes, lose their homes and relatives, or migrate to different provinces of Turkey for short or long periods. Along with this traumatic process, the meaning that individuals attribute to the concept of “home” after the disaster and the extent to which their perceptions of this concept have transformed have become an important research topic. In this context, the current study examines in detail the transformation in the thoughts of individuals aged 18–24 who directly experienced the Kahramanmaraş Twin Earthquakes of February 6, 2023 in Malatya regarding the concept of home. For this purpose, the following research questions were sought:

R1: What kind of transformation occurred in the perceptions of young individuals who experienced the February 6, 2023 twin earthquakes regarding the concept of home after the disaster?

R2: What is the effect of the February 6, 2023 earthquakes on the place attachment of young individuals?

It is anticipated that the results of this research will make significant contributions to the development of post-disaster social reconstruction, psychosocial support services and housing policies.

## 3 Methodology

### 3.1 Research design

This research was designed according to a phenomenology design of the qualitative research method. Phenomenology is a research approach that focuses on discovering the common and fundamental structure of the experiences of individuals, that is, the essence of these experiences (Creswell and Creswell, 2018). This method provides an in-depth analysis of personal meanings, emotions, and individual lives. The reason for choosing phenomenology in the current study is to understand the essence of the participants’ perceptions of the concept of home before and after the February 6, 2023 Kahramanmaraş earthquakes within the framework of place attachment.

### 3.2 Study group

The study group of the research consists of 95 participants who directly experienced the earthquake centered in Kahramanmaraş on February 6, 2023 in Malatya Province. 50 of the participants were female and 45 were male, and their ages ranged from 18 to 24. In determining the research participants, the criterion sampling method, which is frequently preferred in qualitative research, was used. In this direction, the basic criteria sought in the participants included in the study group were determined as; the participants having directly experienced the earthquake, the houses they lived in being heavily damaged or completely destroyed during the earthquakes. In this way, the study group was ensured to consist of individuals who had meaningful experiences in terms of concepts such as post-earthquake home perception and place attachment.

### 3.3 Data collection

Data were collected through two open-ended questions focused on understanding the participants’ perceptions of the concept of “home” before and after the disaster. Participants were asked the questions “What did home mean to you before the earthquake?” and “What does home mean to you after the earthquake?” and were asked to express their responses in writing. The main purpose of this method is to allow participants to express their individual lives, experiences and personal meanings freely and without restriction. The participants’ written responses were transcribed into the text as is without any intervention by the researchers.

### 3.4 Analysis of data

The data were analyzed using a thematic analysis approach. In the first stage, participants’ written responses were transcribed in full. Then, two researchers independently read the data in detail and identified meaningful units; these units were converted into open codes according to similarities in their content. Following the coding process, the relevant codes were discussed among the researchers and combined under supercategories, and the categories were organized in a comparative structure under the headings of “pre-earthquake home perception” and “post-earthquake home perception”. The obtained data were supported by direct quotes to strengthen the credibility of the findings. ChatGPT o3 (version May 2025) was also used to make the obtained data more visually readable (Şen et al., 2023).

### 3.5 Validity and reliability

In this study, most of the strategies suggested by Creswell and Poth (2016) to increase the validity and reliability in qualitative research were implemented. In the first stage of the analysis process, two researchers independently examined each participant’s text, created preliminary codes, and identified the main themes emphasized in the texts. In the second stage, the researchers came together and compared the preliminary codes, created a common code list by consensus, and grouped similar codes into subcategories in terms of content. In the following step, similar subcategories were combined under main categories, taking into account thematic integrity.

To enhance the clarity and consistency of the presentation of codes, subcategories, and categories, the artificial intelligence-supported language model ChatGPT o3 was used in a limited and supportive manner during the writing process. Its use was restricted to language and text organization purposes, including wording consistency, clarity of expression, and the presentation of theme titles. All analytic decisions, interpretations, coding procedures, and thematic structuring remained the responsibility of the researchers. In addition to researcher consensus, comparative checks were carried out to review the coherence of the wording used in the themes and subthemes (Şen et al., 2023). During the analysis, the researchers who experienced uncertainty in the content of some texts asked additional questions to the relevant participants and obtained partial participant feedback (member checking) and confirmed the accuracy of the data. Throughout the process, the constant comparison technique was applied and the codes, categories and themes were reviewed; the credibility of the results was strengthened with descriptive quotes. In addition, the researchers’ first-hand experience of the earthquake allowed them to interpret the participant experiences with a deeper empathic understanding, which contributed to the holistic and meaningful analysis of the data. The researchers kept reflexive notes, constantly questioned the possible effects of subjective perspectives on the analysis, and clarified the decision-making processes through joint discussions, thus increasing the internal validity and reliability of the study.

## 4 Results

**Figure 1:**
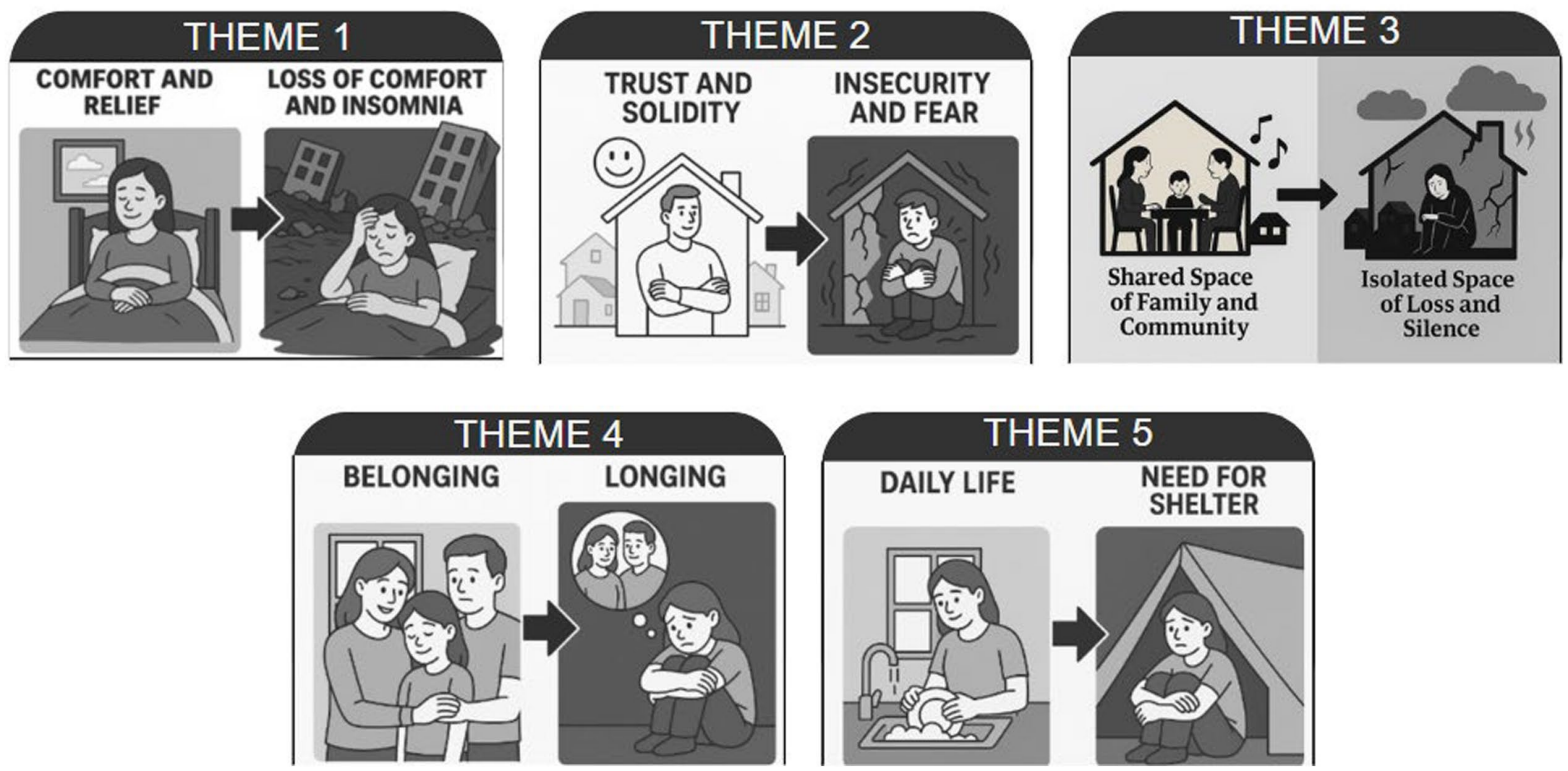
Themes of change after the earthquake.

Participants’ perceptions of home differed significantly in two periods, before and after the earthquake. As a result of the thematic analysis, five main theme pairs were identified: “Comfort and Relief→Loss of comfort and insomnia”, “Trust and Solidity→Insecurity and Fear”, “Shared Space of Family and Community→Isolated Space of Loss and Silence”, “Belonging→Longing”, “Daily Life→Need for Shelter”. Each theme pair is presented below in a comparative manner, with statements from before and after. Each theme pair clearly expresses the mental schemas before the earthquake, the ruptures after the earthquake, and the shifts in meaning between the two (Figure 1).

### 4.1 Comfort and relief → loss of comfort and insomnia

Before the earthquake, home was described as a space where participants could withdraw from the demands of daily life and recover both physically and emotionally. It functioned as a private inner world shaped by familiarity, routine, and comforting objects. In this sense, home was not simply a shelter. It was also a place where psychological balance could be maintained and everyday life could continue in a stable way.

“*Daily fatigue, stress, pain, joy and all these emotions evaporate in front of the door and I enter my harbor*.” P32

“*Before the earthquake, home was a place I would return to. Wherever I went I could turn back. I saw it as a place of relaxation. It was the place in the world where I felt most comfortable.*” P25

These statements show that comfort was experienced in more than one way. It referred to physical rest, but it also carried emotional and psychological meanings. Home was associated with release, privacy, and the possibility of feeling without restraint. Participants described it as a place where the self could settle and where external pressures could be left behind. After the earthquake, this sense of comfort was deeply disturbed. The house no longer appeared as a site of ease and restoration. Instead, it became associated with fear, vigilance, and uneasiness.

“*I don’t feel safe like I used to. The warmth, comfort, peace, everything is gone. If possible, I would move into a caravan.*” P3

“*After the earthquake, I want the old home environment but I can’t find the comfort I’m looking for. Because it’s a reason for fear for me…*” P73

“*Going to sleep with prayers at night and waking up suddenly with aftershocks is a situation that really stresses people out.”* P43

“*After the earthquake, I did not want to enter the house. I distanced myself from home a great deal. Home started to feel like a very bad place to me. When I was scared I would retreat to the house but after the earthquake the situation changed and when I was scared I would go out*.” P75

These post-earthquake statements do not reflect a single form of discomfort. They point to different patterns of rupture in the experience of home. In some accounts, discomfort appears as a lasting sense of alienation from the former home atmosphere. P73, for example, expresses an ongoing inability to recover the emotional texture of the old home. The loss here is not limited to one frightening moment. It reflects a more continuous estrangement from what home once meant. In other accounts, discomfort takes a more bodily and cyclical form. P43 describes a repeated night-time experience shaped by prayer, aftershocks, and sudden awakening. This suggests not only fear, but also an enduring condition of alertness that interrupts sleep and prevents rest. A further shift appears in P75’s statement. Here, the house no longer functions as a refuge to move toward in moments of fear. It becomes a place to move away from. This reversal is important because it shows that the protective meaning of home has been fundamentally altered.

This transformation can be read through the emotional dimension of place attachment. In the context of Scannell and Gifford (2010) tripartite model, the positive feelings once associated with home were replaced by anxiety, threat, and instability. Yet the findings suggest more than the simple disappearance of comfort. They show that comfort itself was redefined after the earthquake. What was once experienced as relief, safety, and emotional settling became tied to vigilance, interrupted sleep, and fear-based withdrawal. In this sense, comfort emerges not as a purely physical condition, but as a relational and perceptual experience grounded in trust. Once that trust was broken, home could no longer be inhabited in the same way.

### 4.2 Trust and solidity → insecurity and fear

Before the earthquake, home was described by participants as the safest place in their lives. Thick walls, familiar rooms, and known objects created a sense of physical protection and emotional stability. In this sense, safety was tied to the building itself and to the continuity and predictability it represented.

*“It seemed like it would always stay in place and never shake.”* P11

“*Just as a baby feels safe and secure in its mother’s arms, for me the house was also a source of peace and security.*” P29

These statements overlap with Proshansky (1978) concept of place identity, reflecting how the individual’s self-perception integrates with the physical environment. In this respect, home is not only an objective structure; it is also the place of the individual’s emotional security. However, after the earthquake, this sense of security was dramatically damaged; the house was now perceived as a threatening place, devoid of physical and emotional security. According to the participants, every structural element of the house was considered a potential risk, and anxiety was intense, especially at night. At the same time, the participants did not want to stay in the house and had doubts about the physical strength of the structure.

“*It has become a place to be afraid of. A place where we have no security and always live in fear of it collapsing on us. Are the columns solid?*” P94

“*I don’t feel safe at home anymore. I used to try to stay at home more. Now I don’t want to stay inside the house for more than five minutes even when I need something.*” P46

“*It is the place where I lost my sense of trust. I am confronted with the painful reality that it could be taken away from me at any moment. The place where I lost my sense of trust. My home, my safe haven, is now like a temporary inn door.*” P40

“*Again, I don’t trust buildings. Houses are buildings that we spend a lot of time in, and I don’t trust them structurally.*” P45

“*The house that is the most sheltered place for me now gives me the feeling that it will turn into my grave with the slightest shake or sound.*” P64

These statements point to different forms of insecurity. In some accounts, fear appears as structural doubt. Participants question the strength of columns, walls, and the building’s ability to stand. In others, insecurity takes the form of constant mental anticipation. P62 is especially striking in this respect. The metaphor of the house as a “soft cookie” suggests a complete loss of structural solidity, while the image of the wall falling on one’s head turns fear into something immediate, bodily, and vividly imagined. The house is no longer only seen as unsafe. It is imagined as an environment that can suddenly injure or kill. Some participants express this rupture through avoidance. P46, for example, shows that the loss of trust is strong enough to limit the time spent inside the house. In other statements, insecurity becomes more symbolic and existential. P40 suggests that home has lost its meaning as a safe haven, while P64 presents the house as a possible grave. This is important because it shows that fear is not limited to physical danger. It reshapes the meaning of home at emotional, symbolic, and bodily levels.

This transformation can be understood through Scannell and Gifford (2010) tripartite model of place attachment, especially in terms of the disruption of both environmental and emotional trust. What was once experienced as solidity and protection gave way to watchfulness, doubt, and anticipatory fear. In this new condition, home no longer represents existential security. It becomes a place where collapse is imagined, safety is questioned, and everyday inhabitation is shadowed by the possibility of destruction.

### 4.3 Shared space of family and community → isolated space of loss and silence

Home carried meaning through shared life as much as through personal experience. Participants described it as a place shaped by family gatherings, conversations, meals, neighborhood encounters, and ordinary moments of togetherness. In this sense, home was remembered as a social environment where emotional closeness and everyday continuity were sustained.

“*Before, for me, home was a place where the whole family met, chatted and shared their troubles”* P6

“*While, home was a family Sunday breakfast…“*P14

“*When I come back from school or anywhere, it is a door where many dreams pass through in my mind. For example, “What did my mother cook?” Seeing my neighbors every morning made me happy.*” P43

These statements show that the meaning of home was closely tied to routine forms of sociality. Home emerged as a lived space shaped by family intimacy, neighborhood familiarity, and shared memory. This aligns with Twigger-Ross and Uzzell (1996) view that environmental identity develops through relationships as well as personal experience. In our data, home was a place where people recognized themselves through others and through repeated forms of daily interaction. After the earthquake, this collective aspect was seriously shaken; memories of home turned into a traumatic void with broken ties. Participants stated that the sharing at home was lost and that what was experienced together was now only in the past.

“My room, while it was my confidant, became a place that bothered me to the point of wondering if it would be my grave, and I never felt the feather comfort of that bed again.” P34

“*OUR NEIGHBORHOOD IS DESTROYED. Dreams are also destroyed.”* P21

“*The houses where we gathered with sincere friends and had fun became the stops where we waited for our deaths as if we were in coffins*.” P36

“*My education life became stagnant. I stayed away from my school and friends for a long time. Even my friendships weakened. I actually wanted to retreat into solitude.*” P43

“*I lost many people I loved. Our future, our friendships, our lives and every corner where we had accumulated our memories were destroyed.*” P90

These statements reveal different forms of social loss. In some accounts, the rupture appears through the collapse of intimate domestic comfort. P34 shows how a room once associated with trust and emotional closeness became a source of fear and unease. In other accounts, loss is expressed at the scale of the neighborhood. P21 condenses the destruction of place and future into a single image, suggesting that the collapse of the built environment also shattered shared expectations and communal continuity. P36 presents an even sharper reversal. A house that had once held friendship and joy is now imagined as a coffin-like waiting place. This shows how spaces of sociability can be transformed into spaces of collective dread. P43 points to another layer of loss. Here, the rupture extends beyond the house itself and reaches school life, friendship, and the willingness to remain socially connected. P90 brings these layers together by linking personal grief, broken relationships, and the destruction of memory-laden places.

Taken together, these losses show that the earthquake damaged the collective dimension of environmental memory. Home no longer appears as a place where social ties are renewed and everyday togetherness is sustained. It becomes a space marked by absence, broken continuity, and silence. In this sense, the loss of home involves more than the destruction of walls or rooms. It includes the collapse of shared rhythms, familiar relationships, and the social worlds that once gave home its emotional depth.

### 4.4 Belonging → longing

The participants’ sense of belonging to their homes provide important data within the scope of place identity theory. In this context, the home functions as an extension of the individual’s self within the framework of Proshansky (1978) place identity theory. The sense of belonging at home is based not only on physical ownership but also on the time spent there and the experiences gained.

*“Home is a home, a place where you feel like you belong somewhere, where you hold on to life and your family behind its doors.”* P87

“*It was the only place where I felt safe and secure. It was a place where I felt like I belonged.*” P46

“*The house gave me a feeling of belonging.*” P59

“*When I think about the concept of home, for me it was a haven of refuge, a peaceful environment, a private space where I could listen to myself.*” P23

“*My home was always precious to me. I loved and missed the warmth of my home without noticing the changes over time, but always without losing the sense of belonging.*” P8

These statements show that belonging was experienced as emotional rootedness. Home was described as a place where participants felt held, recognized, and connected to life. In this sense, belonging was tied to continuity, familiarity, and the quiet certainty of having a place in the world. After the earthquake, this bond was deeply shaken. Participants no longer described home as a place where they belonged. Instead, home appeared as something distant, unreachable, or emotionally lost. In this shift, longing emerged as a central response to the disappearance of belonging.

“*I missed the sound of plates being prepared for dinner, the sounds of the song I turned on early in the morning, and running in our hallway since I was little. Home became a longing for me. I missed the feeling of belonging.*” P8

*“Now, even though I want to trust, I can’t find the sense of belonging that I felt in my old home.”* P18

*“I felt lost. But the feeling inside me was not that I had a place to shelter, but that the environment where I had different memories in every corner had suddenly disappeared.*” P59

These statements point to different forms of longing. In P8, longing is tied to sound, routine, and childhood memory. What is missed is not only the house itself, but the sensory and emotional world once attached to it. P18 reflects a different experience. Here, the desire to trust remains, but the old sense of belonging cannot be recovered. This suggests an ongoing gap between the wish to reconnect and the inability to feel at home again. P59 expresses loss through disorientation. The speaker does not focus on the absence of shelter, but on the disappearance of a memory-filled environment. This is important because it shows that longing is directed not simply toward a building, but toward a lived world that has collapsed.

This transformation reflects the disruption of place identity through trauma. The earthquake did not only distance individuals from their homes in a physical sense. It also disrupted the subjective experience of belonging that made home feel like part of the self. In this new condition, home is remembered less as a place of present attachment and more as a site of absence, memory, and unresolved longing.

### 4.5 Daily life→ need for shelter

Home was described by participants as a place where ordinary life could unfold without interruption. It was the setting of habits, small routines, family roles, and everyday comfort. In this sense, the meaning of home was closely tied to the unnoticed rhythm of daily life.

“*It was such a commonplace phenomenon for me that I didn’t remember how I felt when it came to home before the earthquake.*” P44

*“I see it as taking care of the housework of my mother and father due to their old age. Peaceful but tiring. Very complicated. There are many things in the house. There was...”* P1

“*It was the place where I opened the fridge door whenever I wanted, ate food whenever I wanted, took a hot shower whenever I wanted and lay on the couch without any worries.*” P2

These recurring experiences are the constitutive elements of emotional stability, along with environmental memory. The home acts as an anchor that reinforces the individual’s sense of control. After the earthquake, this daily structure was interrupted, and the home was transformed in terms of both time and function. The home has now become a place of memory where daily routines are forgotten or an idealized refuge; material elements that were once important have lost their importance.

“*I saw that the smaller the better. I saw that it doesn’t really matter where and how the home is.” P53*

*“The place where you spend the night is your home. Home has become a verb rather than a concept. Even if it’s not an object, it’s a home... It’s a place where you take shelter*” P1

“*It turns out that the walls I thought would never be shaken could be shaken and collapsed without me moving in. I learned that the things I had taken for granted could disappear in an instant.*” P2

“*Although people approached their home environment and order with a different logic (decoration, ostentation, etc.) before the earthquake, after the earthquake they began to better understand how unnecessary and empty space such things take up in people’s lives.*” P66

“*I forgot where my father lived in the home or where he smoked. I forgot how my mother spent a day in this home.*” P95

These statements point to different forms of functional and symbolic change. P53 reflects a move away from size, comfort, and spatial form toward minimal safety and bare necessity. P1 goes further by redefining home itself. The statement that “home has become a verb” suggests that home is no longer understood as a stable place or object, but as a temporary act of sheltering. P2 expresses a collapse in taken-for-granted continuity. What had once seemed fixed and unquestionable is now understood as fragile and temporary. P66 introduces a change in values. Decoration and display lose importance, while usefulness and survival come to the foreground. P95 reveals another kind of rupture. Here, the loss is tied to domestic memory. The speaker no longer remembers how family members inhabited the house in everyday life, which suggests a fading of intimate spatial knowledge.

Overall, the findings show that the earthquake suspended the routine-based role of home as a source of peace, continuity, and control. In its place, a narrower and more fragile meaning emerged. Home came to be understood less as a setting for everyday life and more as a temporary space of shelter. At the same time, it retained traces of memory and loss. In this sense, the post-earthquake home appears as a space shaped by survival, absence, and the remains of ordinary life.

## 5 Discussion

This research shows that the meaning that young people living in Malatya attributed to the concept of “home” after the twin earthquakes centered in Kahramanmaraş on February 6, 2023 entered a deep reconstruction process on spatial, emotional and social levels. Home, which was identified with comfort, privacy, security, memories and family before the disaster, evolved into a place where trauma, uncertainty and a chronic perception of risk materialized after the disaster. The findings also reveal that the “process-emotional” dimension in Scannell and Gifford (2010) three-component place attachment model was reversed, and feelings of security and peace turned into anxiety, fear and insomnia.

Taken together, however, these findings suggest more than a weakening of an existing person–place bond. They indicate that the earthquake constituted a rupture in the lived meaning of home itself. Before the disaster, home was largely encountered as a taken-for-granted site of routine, familiarity, protection, and emotional continuity. After the disaster, it became an ambivalent and internally contested space shaped simultaneously by fear, grief, memory, and the struggle to restore meaning. In this sense, the contribution of the present study is not to reject established place attachment models, but to extend them by showing that, in post-disaster contexts, attachment is not simply reduced. Rather, it is reconfigured as a fragile, trauma-inflected, and phenomenologically transformed experience of belonging.

The person-place bond certainly involves emotional attachment to a particular place. Place attachment can be understood as an emotional bond with an environment that responds to a basic human need for security, familiarity, and belonging (Relph, 1976). In our data, the pre-earthquake perception of home was closely associated with peace, privacy, warmth, memories, and family. In this sense, home stood at the center of participants’ emotional attachments, place identity, and social belonging. This finding is consistent with earlier studies that emphasize the role of place in the construction of identity (Proshansky, 1978; Peng et al., 2020). However, the post-earthquake narratives, as also noted in previous research on disaster-affected housing perception (Asadi et al., 2021), reveal a sharp break in how home was experienced. A place once associated with safety, warmth, and belonging came to be linked with anxiety, uncertainty, fear, and physical risk. In Scannell and Gifford (2010) tripartite model of place attachment, this transformation is especially visible in the process dimension, particularly at the emotional level. Feelings of comfort, peace, and protection gave way to insomnia, vigilance, and loss of ease. This suggests not simply a weakened attachment, but a profound change in the emotional conditions through which attachment is lived. This shift highlights the boundaries of traditional structural frameworks, which often assume a continuous and stable person-place bond. By adopting a phenomenological orientation, this study demonstrates that in the wake of disaster, attachment is less a static outcome and more a deeply reflexive and internalized process.

Before the earthquake, home was experienced as a safe and emotionally stabilizing environment that supported the feeling of being at home. After the disaster, it became associated with uncertainty, bodily alertness, fear, and physical risk. This shift is consistent with earlier findings on post-disaster changes in housing perception and residential satisfaction (Asadi et al., 2021; Kürüm Varolgüneş, 2021). Still, our findings suggest more than a loss of comfort. Security is central to the formation and maintenance of place attachment (Scannell and Gifford, 2010). When trust in the dwelling collapses, the basis of attachment changes as well. In this sense, the house no longer serves as a secure anchor of everyday life. It turns into a precarious space where attachment, if it continues at all, is shaped by vulnerability. This transformation can also be compared with post-disaster housing studies. Tas et al. (2007), in their evaluation of permanent housing in Kocaeli-Gündoğdu, showed that post-earthquake housing should not only provide shelter but also respond to survivors’ psychological, social, and economic expectations. Their findings further suggest that residents’ evaluations are shaped by safety perception as well as the broader housing environment, including social and physical conditions. In this respect, our findings extend the post-disaster housing literature by showing that when trust in the dwelling is disrupted, the problem is not limited to reduced satisfaction. Rather, home itself may lose its function as an emotionally stabilizing place and become a space associated with fear, uncertainty, and vulnerability.

A strong sense of belonging often develops through close social ties, intergenerational roots, neighborhood familiarity, and shared daily rituals (Lewicka, 2011; Twigger-Ross and Uzzell, 1996). In this study, the pre-earthquake home created a vivid socio-spatial memory through family breakfasts, brief conversations with neighbors, and evening gatherings with friends. After the disaster, home was no longer remembered only as a site of intimacy. It also emerged as a space of loss. Participants described collapsed houses and neighborhoods with expressions such as coffin, grave, and demolished neighborhood. This shows that the earthquake damaged not only the material structure of housing but also the relational and ritual dimensions through which home had been lived and remembered. This relational rupture is consistent with post-disaster housing research from Türkiye (Kürüm Varolgüneş, 2021; Şeremet et al., 2024; Tekin Babuc and Gokce, 2024). In their study of women’s experiences in post-disaster recovery housing after the 2011 Van earthquake, Şeremet et al. (2024) showed that housing should be understood as a setting that shapes social networks, daily practices, and lived vulnerability. Their findings suggest that relocation can intensify social dislocation by weakening existing neighborhood ties and making adaptation to new residential environments more difficult. In a similar way, the present study shows that the loss of home after the earthquake was not merely material. It involved the collapse of familiar routines, shared memories, and everyday forms of togetherness through which home had previously been lived as a place of belonging. Recovery, therefore, requires more than physical reconstruction. Studies on socio-spatial reconnection suggest that collective rituals, neighborhood interaction, and shared commemorative practices may help restore environmental memory and rebuild the bond between home, emotion, and community (Berroeta et al., 2024; Reza et al., 2024).

Place identity refers to the way individuals build a sense of belonging through their relationship with place and through the meanings that accumulate in lived environments (Stedman, 2002). People attach meaning to places through memory, and these meanings often become part of their core identities. Places associated with unforgettable moments or important life events can create a strong connection between the self and the environment (Scannell and Gifford, 2017). Our findings suggest that the earthquake deeply disrupted this process. Home, which had once been part of participants’ sense of self through routine, familiarity, and emotional continuity, became a symbol of lost belonging and damaged collective memory. In this respect, the disaster did more than destroy buildings. It weakened the identity-supporting role of place. This finding is consistent with studies showing that disasters and forced displacement can erode spatial belonging and turn home into an object of longing tied to the past (Zheng et al., 2019; Carroll et al., 2009). More broadly, sudden destructive events such as earthquakes can weaken individuals’ sense of belonging to both living spaces and social groups, with long-term consequences for psychosocial adaptation and social integration (Ren et al., 2020).

Our findings also show that the psychosocial balance supported by the ordinariness of home and daily routines was suddenly and deeply disrupted by the earthquake. Home usually gains meaning through the rituals and routines of everyday life, and these repeated patterns support emotional stability and a sense of control (Babacan, 2021). When place is lost, what disappears is physical space, personal belongings, social memory, and the socio-spatial routines that structure daily life (Scannell et al., 2016). After the earthquake, the function of home changed radically. The house was no longer experienced as an ordinary living space. It became a strategic survival space shaped by anxiety, preparedness, and implicit escape plans. In this new condition, home lost its quality as a daily living environment and was reduced to the fragile function of a temporary shelter. In line with Asadi et al. (2021), old domestic habits and routines came to be remembered almost like lost objects. This transformation shows that the earthquake changed the lived function of home itself and reshaped belonging and stability at both individual and collective levels.

## 6 Conclusion

This study has shown that the perceptions of young individuals living in Malatya changed radically in spatial, emotional, and social terms following the February 6, 2023, earthquakes. The findings suggest that the disaster did more than produce physical destruction. It disrupted the lived meaning of home and transformed it from a taken-for-granted site of security into an internally contested trauma space. In this sense, the study extends existing understandings of place attachment by showing that, in post-disaster contexts, attachment is not simply weakened. It is reconfigured as a fragile, reflective, and trauma-inflected experience of belonging shaped by memory, loss, and vulnerability. Accordingly, recovery should be understood as more than housing repair. It also requires community-based efforts that help restore collective memory, everyday social life, and the fragile conditions through which belonging can be rebuilt.

## Data Availability

The original contributions presented in the study are included in the article/supplementary material, further inquiries can be directed to the corresponding author.
